# The Clinical and Economic Impact of Point-of-Care CD4 Testing in Mozambique and Other Resource-Limited Settings: A Cost-Effectiveness Analysis

**DOI:** 10.1371/journal.pmed.1001725

**Published:** 2014-09-16

**Authors:** Emily P. Hyle, Ilesh V. Jani, Jonathan Lehe, Amanda E. Su, Robin Wood, Jorge Quevedo, Elena Losina, Ingrid V. Bassett, Pamela P. Pei, A. David Paltiel, Stephen Resch, Kenneth A. Freedberg, Trevor Peter, Rochelle P. Walensky

**Affiliations:** 1 Medical Practice Evaluation Center, Massachusetts General Hospital, Boston, Massachusetts, United States of America; 2 Division of Infectious Diseases, Massachusetts General Hospital, Boston, Massachusetts, United States of America; 3 Division of General Medicine, Massachusetts General Hospital, Boston, Massachusetts, United States of America; 4 Instituto Nacional da Saùde, Maputo, Mozambique; 5 Clinton Health Access Initiative, Maputo, Mozambique; 6 Desmond Tutu HIV Centre, Institute of Infectious Diseases and Molecular Medicine, and Department of Medicine, University of Cape Town Faculty of Health Sciences, Cape Town, South Africa; 7 Harvard University Center for AIDS Research, Harvard Medical School, Boston, Massachusetts, United States of America; 8 Department of Orthopedic Surgery, Brigham and Women's Hospital, Boston, Massachusetts, United States of America; 9 Department of Health Policy and Management, Yale School of Public Health, New Haven, Connecticut, United States of America; 10 Center for Health Decision Science, Harvard School of Public Health, Boston, Massachusetts, United States of America; 11 Department of Health Policy and Management, Harvard School of Public Health, Boston, Massachusetts, United States of America; 12 Division of Infectious Diseases, Brigham and Women's Hospital, Boston, Massachusetts, United States of America; Centers for Disease Control and Prevention, United States of America

## Abstract

**Background:**

Point-of-care CD4 tests at HIV diagnosis could improve linkage to care in resource-limited settings. Our objective is to evaluate the clinical and economic impact of point-of-care CD4 tests compared to laboratory-based tests in Mozambique.

**Methods and Findings:**

We use a validated model of HIV testing, linkage, and treatment (CEPAC-International) to examine two strategies of immunological staging in Mozambique: (1) laboratory-based CD4 testing (LAB-CD4) and (2) point-of-care CD4 testing (POC-CD4). Model outcomes include 5-y survival, life expectancy, lifetime costs, and incremental cost-effectiveness ratios (ICERs). Input parameters include linkage to care (LAB-CD4, 34%; POC-CD4, 61%), probability of correctly detecting antiretroviral therapy (ART) eligibility (sensitivity: LAB-CD4, 100%; POC-CD4, 90%) or ART ineligibility (specificity: LAB-CD4, 100%; POC-CD4, 85%), and test cost (LAB-CD4, US$10; POC-CD4, US$24). In sensitivity analyses, we vary POC-CD4-specific parameters, as well as cohort and setting parameters to reflect a range of scenarios in sub-Saharan Africa. We consider ICERs less than three times the per capita gross domestic product in Mozambique (US$570) to be cost-effective, and ICERs less than one times the per capita gross domestic product in Mozambique to be very cost-effective. Projected 5-y survival in HIV-infected persons with LAB-CD4 is 60.9% (95% CI, 60.9%–61.0%), increasing to 65.0% (95% CI, 64.9%–65.1%) with POC-CD4. Discounted life expectancy and per person lifetime costs with LAB-CD4 are 9.6 y (95% CI, 9.6–9.6 y) and US$2,440 (95% CI, US$2,440–US$2,450) and increase with POC-CD4 to 10.3 y (95% CI, 10.3–10.3 y) and US$2,800 (95% CI, US$2,790–US$2,800); the ICER of POC-CD4 compared to LAB-CD4 is US$500/year of life saved (YLS) (95% CI, US$480–US$520/YLS). POC-CD4 improves clinical outcomes and remains near the very cost-effective threshold in sensitivity analyses, even if point-of-care CD4 tests have lower sensitivity/specificity and higher cost than published values. In other resource-limited settings with fewer opportunities to access care, POC-CD4 has a greater impact on clinical outcomes and remains cost-effective compared to LAB-CD4. Limitations of the analysis include the uncertainty around input parameters, which is examined in sensitivity analyses. The potential added benefits due to decreased transmission are excluded; their inclusion would likely further increase the value of POC-CD4 compared to LAB-CD4.

**Conclusions:**

POC-CD4 at the time of HIV diagnosis could improve survival and be cost-effective compared to LAB-CD4 in Mozambique, if it improves linkage to care. POC-CD4 could have the greatest impact on mortality in settings where resources for HIV testing and linkage are most limited.

*Please see later in the article for the Editors' Summary*

## Abstract 2

Emily Hyle and colleagues conduct a cost-effectiveness analysis to estimate the clinical and economic impact of point-of-care CD4 testing compared to laboratory-based tests in Mozambique.

*Please see later in the article for the Editors' Summary*

## Editors' Summary

**Background:**

AIDS has already killed about 36 million people, and a similar number of people (mostly living in low- and middle-income countries) are currently infected with HIV, the virus that causes AIDS. HIV destroys immune system cells (including CD4 cells, a type of lymphocyte), leaving infected individuals susceptible to other infections. Early in the AIDS epidemic, HIV-infected individuals usually died within ten years of infection. After effective antiretroviral therapy (ART) became available in 1996, HIV infection became a chronic condition for people living in high-income countries, but because ART was expensive, HIV/AIDS remained a fatal disease in low- and middle-income countries. In 2003, the international community began to work towards achieving universal ART coverage, and by the end of 2012, 61% of HIV-positive people (nearly 10 million individuals) living low- and middle-income countries who were eligible for treatment—because their CD4 cell count had fallen below 350 cells/mm^3^ of blood or they had developed an AIDS-defining condition—were receiving treatment.

**Why Was This Study Done?:**

In sub-Saharan Africa nearly 50% of HIV-infected people eligible for treatment remain untreated, in part because of poor linkage between HIV diagnosis and clinical care. After patients receive a diagnosis of HIV infection, their eligibility for ART initiation is determined by sending a blood sample away to a laboratory for a CD4 cell count (the current threshold for treatment is a CD4 count below 500/mm^3^, although low- and middle-income countries have yet to update their national guidelines from the threshold CD4 count below 350/mm^3^). Patients have to return to the clinic to receive their test results and to initiate ART if they are eligible for treatment. Unfortunately, many patients are “lost” during this multistep process in resource-limited settings. Point-of-care CD4 tests at HIV diagnosis—tests that are done on the spot and provide results the same day—might help to improve linkage to care in such settings. Here, the researchers use a mathematical model to assess the clinical outcomes and cost-effectiveness of point-of-care CD4 testing at the time of HIV diagnosis compared to laboratory-based testing in Mozambique, where about 1.5 million HIV-positive individuals live.

**What Did the Researchers Do and Find?:**

The researchers used a validated model of HIV testing, linkage, and treatment called the Cost-Effectiveness of Preventing AIDS Complications–International (CEPAC-I) model to compare the clinical impact, costs, and cost-effectiveness of point-of-care and laboratory CD4 testing in newly diagnosed HIV-infected patients in Mozambique. They used published data to estimate realistic values for various model input parameters, including the probability of linkage to care following the use of each test, the accuracy of the tests, and the cost of each test. At a CD4 threshold for treatment of 250/mm^3^, the model predicted that 60.9% of newly diagnosed HIV-infected people would survive five years if their immunological status was assessed using the laboratory-based CD4 test, whereas 65% would survive five years if the point-of-care test was used. Predicted life expectancies were 9.6 and 10.3 years with the laboratory-based and point-of-care tests, respectively, and the per person lifetime costs (which mainly reflect treatment costs) associated with the two tests were US$2,440 and $US2,800, respectively. Finally, the incremental cost-effectiveness ratio—calculated as the incremental costs of one therapeutic intervention compared to another divided by the incremental benefits—was US$500 per year of life saved, when comparing use of the point-of-care test with a laboratory-based test.

**What Do These Findings Mean?:**

These findings suggest that, compared to laboratory-based CD4 testing, point-of-care testing at HIV diagnosis could improve survival for HIV-infected individuals in Mozambique. Because the per capita gross domestic product in Mozambique is US$570, these findings also indicate that point-of-care testing would be very cost-effective compared to laboratory-based testing (an incremental cost-effectiveness ratio less than one times the per capita gross domestic product is regarded as very cost-effective). As with all modeling studies, the accuracy of these findings depends on the assumptions built into the model and on the accuracy of the input parameters. However, the point-of-care strategy averted deaths and was estimated to be cost-effective compared to the laboratory-based test over a wide range of input parameter values reflecting Mozambique and several other resource-limited settings that the researchers modeled. Importantly, these “sensitivity analyses” suggest that point-of-care CD4 testing is likely to have the greatest impact on HIV-related deaths and be economically efficient in settings in sub-Saharan Africa with the most limited health care resources, provided point-of-care CD4 testing improves the linkage to care for HIV-infected people.

**Additional Information:**

Please access these websites via the online version of this summary at http://dx.doi.org/10.1371/journal.pmed.1001725.

- The World Health Organization provides information on all aspects of HIV/AIDS (in several languages); its “Consolidated Guidelines on the Use of Antiretroviral Drugs for Treating and Preventing HIV Infections: Recommendations for a Public Health Approach”, which highlights the potential of point-of-care tests to improve the linkage of newly diagnosed HIV-infected patients to care, is available
- Information is available from the US National Institute of Allergy and Infectious Diseases on HIV infection and AIDS
- NAM/aidsmap provides basic information about HIV/AIDS, and summaries of recent research findings on HIV care and treatment; it has a fact sheet on CD4 testing
- Information is available from Avert, an international AIDS charity, on many aspects of HIV/AIDS, including information on starting, monitoring, and switching treatment and on HIV and AIDS in sub-Saharan Africa (in English and Spanish)
- The “UNAIDS Report on the Global AIDS Epidemic 2013” provides up-to-date information about the AIDS epidemic and efforts to halt it
- Personal stories about living with HIV/AIDS are available through Avert, Nam/aidsmap, and Healthtalkonline

## Introduction

In sub-Saharan Africa, over 50% of HIV-infected patients remain unlinked to clinical care, despite the dramatic scale-up of HIV treatment over the past decade [1]. Point-of-care technologies have been widely promoted as a mechanism to improve triage and linkage of newly diagnosed HIV-infected patients to care [2]–[5].

After initial HIV diagnosis, patients undergo immunological staging, in which the severity of immunosuppression, as quantified by CD4 count, determines eligibility for antiretroviral therapy (ART) initiation. Current standard of care throughout sub-Saharan Africa at the time of HIV diagnosis is a laboratory-based CD4 test, when available [5]. Patients receive the results of the CD4 test at a return visit and are triaged to clinical care depending on their ART eligibility as determined by national policy guidelines [6]. Patient attrition at each of these steps after HIV diagnosis is high, ranging from 17% to 80% in resource-limited settings [7]–[13]. The World Health Organization (WHO) has targeted these steps in linkage to care as opportunities for improvement and has underscored the potential offered by point-of-care CD4 tests to expedite immunologic staging [5].

Data from multiple regions throughout sub-Saharan Africa demonstrate that point-of-care CD4 testing (POC-CD4) can improve overall linkage to care [14]–[18]. Our objective is to assess the clinical outcomes and cost-effectiveness of POC-CD4 compared to laboratory-based CD4 testing (LAB-CD4) for immunologic staging in Mozambique and to examine the generalizability of these results to settings throughout sub-Saharan Africa with a diversity of opportunities to access care.

## Methods

### Ethics Statement

This work was approved by the Partners Human Research Committee, Boston, Massachusetts, US (Protocol #2003 P001019).

### Analytic Overview

We use the Cost-Effectiveness of Preventing AIDS Complications–International (CEPAC-I) model to project the clinical impact, costs, and cost-effectiveness of POC-CD4 at the time of HIV diagnosis at outpatient voluntary testing and counseling (VCT) clinics [19]–[22]. In a simulated cohort of newly diagnosed HIV-infected patients in Mozambique, we investigate two strategies for immunologic staging: LAB-CD4 versus POC-CD4. The two strategies differ in terms of (1) the probability of linkage to care, (2) CD4 test sensitivity and specificity, and (3) CD4 test cost. For each strategy, the model simulates 2 million patients to produce stable outputs. We use deterministic and probabilistic sensitivity analyses to examine the generalizability of our results to sub-Saharan settings with different populations, infrastructures, and available resources for HIV testing, clinical care, and retention in care.

We use the model to project 5-y survival, life expectancy (LE), and per person lifetime direct medical costs of HIV care (in 2011 US dollars). Future benefits and costs are discounted at 3% per year [23]. We calculate the incremental cost-effectiveness ratio (ICER) as the ratio of the difference in outcomes between the two strategies of care (Δcosts/ΔLE) after immunologic staging [23]. Guided by recommendations from the WHO Commission on Macroeconomics and Health [24] and WHO-CHOICE [25], we consider a strategy to be “cost-effective” if its ICER is less than three times the country-specific per capita gross domestic product (GDP) (Mozambique 2011 GDP, US$570) and “very cost-effective” if its ICER is less than one times the per capita GDP [26].

### Model Structure

The CEPAC-I model is a previously published Monte Carlo state-transition model of HIV natural history, case detection, linkage, and treatment [19]–[22].

#### Cohort characteristics

At the simulation's start, patients have just been diagnosed with HIV at a VCT clinic. Their baseline characteristics are drawn randomly from distributions of age, gender, CD4 count, and HIV RNA, populated from region-specific clinical trials and cohort data [14],[27]. In each month, simulated patients move between health states, specified broadly as chronic HIV infection, acute clinical events, and death. The simulated cohort excludes incident and acute HIV cases.

#### Linkage to care

**Figure 1:**
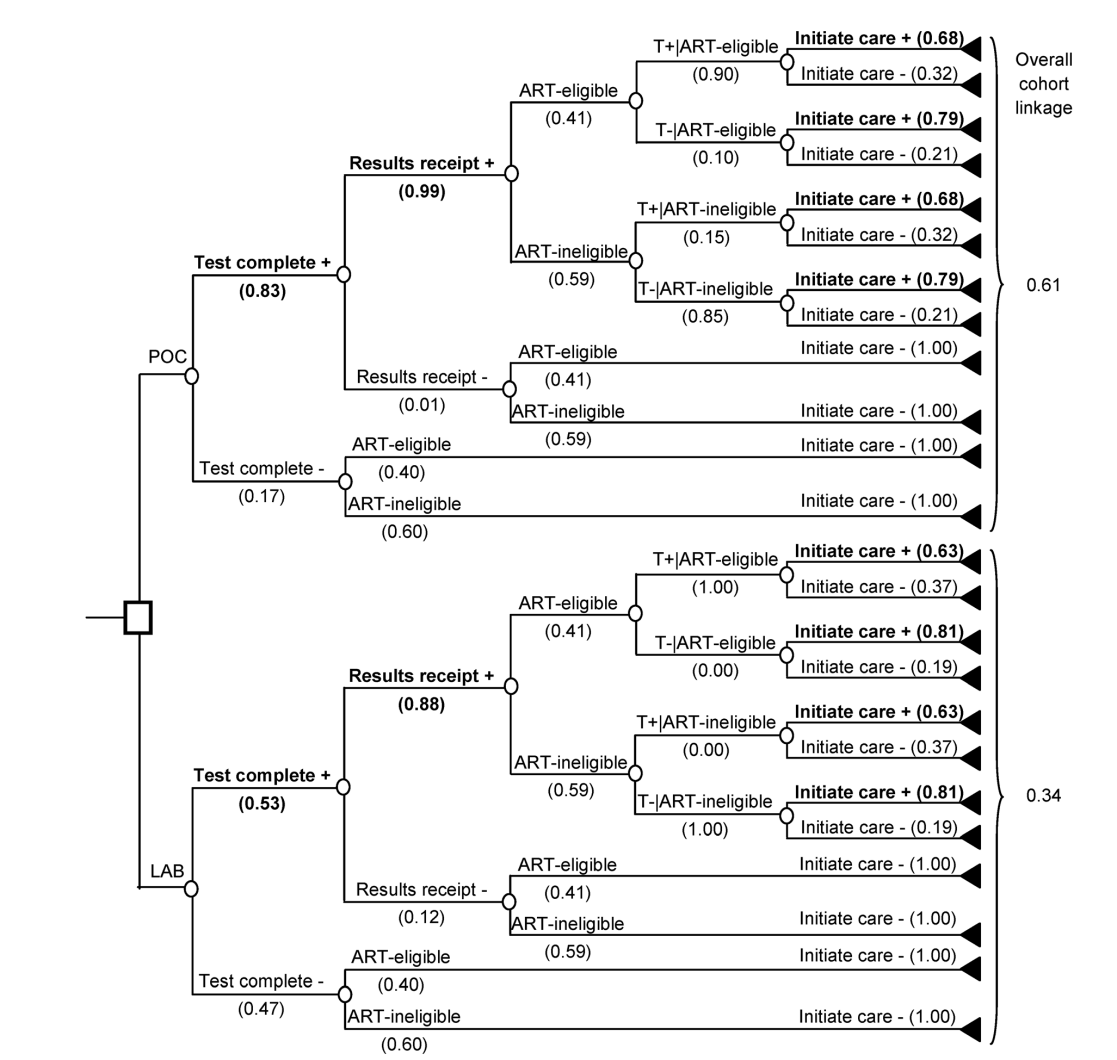
Schematic comparing two strategies for immunological staging after HIV diagnosis: LAB-CD4 and POC-CD4. In both LAB-CD4 and POC-CD4, literature-based probabilities are shown for: completing a laboratory CD4 test, receiving the test result, and successful initiation of care (Table 1). Probability of linkage depends on ART eligibility as determined by the “true CD4 count,” as well as the “observed CD4 count” test result (T) [14]. Bolded branches denote where the outcomes result in linkage to care. T+, observed CD4 count ≤250/µl; T−, observed CD4 count>250/µl.

To link to care after HIV diagnosis, simulated patients must complete a CD4 test for immunological staging, which determines ART eligibility. Linkage to care requires completion of three sequential steps: (1) obtaining the patient sample and completing the CD4 test (“test completion”), (2) alerting the patient to the test result (“result receipt”), and (3) initiating HIV clinical care after receipt of the immunologic staging result (“initiating care”). Based on the “true CD4 count” (in vivo), patients are either “ART eligible” (i.e., true CD4 count ≤ threshold for ART eligibility) or “ART ineligible” (i.e., true CD4 count> threshold). The “observed CD4 count” is the test result given to the patient, which can differ from the true CD4 count depending on CD4 test characteristics. Patients can receive observed CD4 count results 1 wk or more after HIV diagnosis (LAB-CD4) or sooner (POC-CD4). For patients who receive their CD4 test result, the probability of initiating care depends on the observed CD4 count (Figure 1). Patients who do not initiate care after immunologic staging (i.e., “unlinked”) can link in subsequent months following an acute opportunistic infection (OI), tuberculosis (TB), or if they undergo repeat HIV testing or immunologic staging. Unlinked patients progress with natural history of HIV.

#### CD4 count for immunologic staging

We use two parameters to calculate the observed CD4 count compared to the true CD4 count: precision and bias. The coefficient of variation (CV) characterizes precision (i.e., the variability of observed CD4 count from repeat tests on a single specimen); we calculate the observed CD4 count as the true CD4 count plus a random draw from a normal distribution with zero mean and standard deviation (SD) equal to the product of the CV and the true CD4 count. To represent any systematic bias of the CD4 test (i.e., if the observed CD4 count is consistently greater or less than the true CD4 count), we also vary the mean of the observed CD4 count from the true CD4 count. For both precision and bias, we use a percentage of the true CD4 count because variations in the observed CD4 count will range more widely for higher true CD4 count values [28].

CD4 test sensitivity and specificity depend on three parameters: the test's precision and bias, the true CD4 count of the population, and the ART-eligibility threshold. We use model output for true and observed CD4 counts to calculate the sensitivity and specificity associated with an ART-eligibility threshold.

Patients whose ART eligibility is misclassified by the point-of-care CD4 test are triaged and treated accordingly. If misclassified as ART eligible (i.e., truly ART ineligible, with true CD4 count>250/µl but observed CD4 count ≤250/µl), linked patients initiate ART, which improves their projected clinical outcomes and increases costs. Patients misclassified as ART ineligible (i.e., truly ART eligible, with true CD4 count ≤250/µl but observed CD4 count>250/µl) do not start ART after linkage and have worse clinical outcomes. To remain conservative towards potential benefits of POC-CD4, patients initially misclassified as ART ineligible are never successfully transitioned to ART.

#### HIV clinical care

Linked patients are treated with care concordant with national guidelines in Mozambique according to the observed CD4 count, including clinical visits, laboratory monitoring, and ART for eligible patients. ART efficacy depends on patient adherence; those with better adherence have a greater probability of virologic suppression and associated immunologic recovery [29]. To account for interruptions in care after linkage [30]–[32], simulated patients have a monthly probability of becoming lost to follow-up (LTFU), with a probability of returning to care. When LTFU, patients discontinue ART and co-trimoxazole prophylaxis and experience HIV natural history.

### Input Parameters

#### Cohort characteristics

We derive cohort characteristics from a published study of immunologic staging in Mozambique: mean CD4 count 300/µl (SD, 230/µl), mean age 32.7 y (SD, 10.1 y), and 65% female (Table 1) [14].

**Table 1:** Model input parameters for analysis of immunological staging by POC-CD4 versus LAB-CD4 in Mozambique.

Category	Variable	Base Case Value		Range (Minimum–Maximum)	Reference
		LAB-CD4	POC-CD4		
**Cohort characteristics**	**Mean CD4 count, cells/µl (SD)**	300 (230)	Same	50–800	[14]
	**Mean age, years (SD)**	32.7 (10.1)	Same	20–70	[14]
	**Female, percent**	65	Same	0–100	[14]
**Immunological staging characteristics**	**Sensitivity, percent** a	100	90	85–100	[36]
	**Specificity, percent** a	100	85	79–100	[36]
	**Overall linkage for cohort, percent**	34	61	10–100	Adapted from [14]
	Test completion, percent	53	83	10–100	Adapted from [14]
	Results receipt, percent	88	99	10–100	Adapted from [14]
	Initiation of care for observed ART-eligible patients, percent	63	68	10–100	Adapted from [14]
	Initiation of care for observed ART-ineligible patients, percent	81	79	10–100	Adapted from [14]
	**CD4 test cost, US dollars**	10	24	10–1,000	[39],[40]
**Range of regional access to HIV care**	**Linkage after WHO stage 3 or 4 OI, percent**	75	Same	100, 50, 25	Assumption
	**Linkage after TB, percent**	43	Same	65, 25, 13	[33],[34]
	**Frequency of routine HIV testing**	Every 10 y	Same	Every 5 y, once, never	Assumption
**ART efficacy after treatment initiation**	**HIV RNA suppressed at 6 mo, overall percent** b	79	Same		[29]
	**Mean monthly CD4 increase on suppressed ART**				
	Initial 8 wk, cells/µl (SD)	67 (17)	Same		[41]
	Monthly increase after 8 wk, cells/µl (SD)	3 (1)	Same		[41]
	**Loss to follow-up probability, monthly percent** c	0.2–1.1	Same	0–1.9	Derived from [32],[43]
	**Mean time spent LTFU, months (SD)** a	31 (27)	Same	0–60	[32]
**Mozambique national treatment policy**	**ART initiation criteria**				
	CD4 count, cells/µl	≤250	Same		[6]
	OI (WHO stage 3 or 4)	Yes	Same		[6]
	TB	Yes	Same		[6]
	**Available ART**				
	First-line ART	AZT + 3TC + NVP	Same		[6]
	Second-line ART	AZT + 3TC + LPV/r	Same		[6]
**Annual costs (US dollars)**	**Routine HIV care for patients with CD4 count ≤250/µl** d	250	Same	30–380	Adapted from [46]
	**Routine HIV care for patients with CD4 count>250/µl** d	160	Same	20–230	Adapted from [46]
	**First-line ART regimen**	120	Same		[50]
	**Second-line ART regimen**	500	Same		[50]

a Model output using cited input parameters.

b Overall suppression will be lower for second-line ART, as poorly adherent patients are more likely to experience ART failure and initiate second-line ART.

c Loss to follow-up includes interruptions in HIV care of at least 12 mo among those HIV-infected patients who are already linked to care and excludes attrition from care due to mortality or transfers to another clinical care site.

d Costs of routine HIV care on first-line ART include direct costs for inpatient and outpatient care related to HIV infection, co-trimoxazole prophylaxis, ART when initiated and any toxicity if it occurs, and laboratory CD4 tests for ongoing immunological monitoring. We exclude costs associated with absence from work or transport to clinics, as neither the MMOH nor other funding sources are responsible for such costs.

3TC, lamivudine; AZT, zidovudine; LPV/r, lopinavir/ritonavir; NVP, nevirapine.

#### Linkage to care

The overall cohort linkage for LAB-CD4 is 34%. This is equivalent to the product of CD4 test completion (53%), CD4 test result receipt conditional on test completion (88%), and initiation of care conditional on receiving results (74%). Because the probability of initiation of care is different for patients whose observed CD4 count makes them ART eligible (63%) or ineligible (81%), it is weighted by the proportion of the linked population that is ART eligible (42%) or ineligible (58%) (Figure 1; Table 1). In POC-CD4, 61% of the cohort links to care, with improved CD4 test completion (83%) and result receipt (99%). Initiation of care occurs in 74%; 68% from the observed ART-eligible patients and 79% from the ART-ineligible patients, weighted by the 46% of the population who are ART eligible and the 54% who are ART ineligible. For rates of initiating care after receipt of an ART-ineligible CD4 test result, we use unpublished data collected in the same study protocol as the ART-eligible linkage rates [14].

For unlinked patients, we estimate that repeat testing occurs at a monthly rate of once every 10 y, as only 39% of HIV-infected individuals are estimated to know their HIV status in South Africa, where HIV testing services are more robust than in Mozambique [11]. Unlinked patients will link to HIV care 43% of the time if ill with TB (reported range, 13%–62%) [10],[33]–[35] and 75% of the time with WHO stage 3/4 OI (Table 1).

#### CD4 count for immunologic staging

LAB-CD4 uses the gold standard laboratory CD4 test, assuming perfect performance characteristics (sensitivity/specificity, 100%/100%) to ensure the analysis is not biased towards POC-CD4.

**Figure 2:**
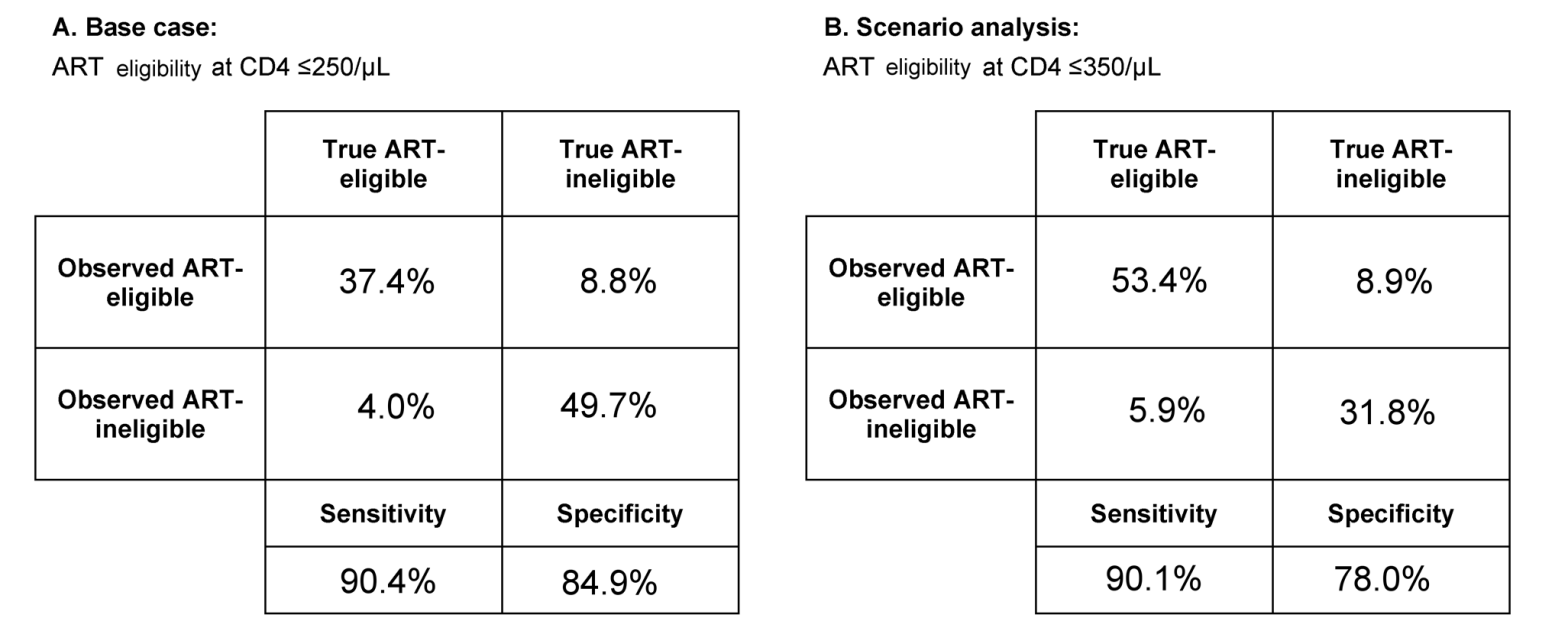
Test characteristics of the point-of-care CD4 test as determined by model output. At the time of HIV diagnosis and immunologic staging, the model captures both the “true CD4 count” of the patient and the “observed CD4 count,” or CD4 test result. The observed CD4 test result has variability around the true CD4 count, depending on the test itself (e.g., point-of-care CD4 test with precision of 32.6% CV). (A) Using model output, we calculate the sensitivity (i.e., observed CD4 count ≤250/µl, true CD4 count ≤250/µl) and specificity (i.e., observed CD4 count >250/µl, true CD4 count >250/µl) for point-of-care CD4 tests when policy sets ART eligibility at CD4 count ≤250/µl. (B) In a scenario in which ART eligibility is at CD4 count ≤350/µl, we use model output to calculate the sensitivity (i.e., observed CD4 count ≤350/µl, true CD4 count ≤350/µl) and specificity (i.e., observed CD4 count >350/µl, true CD4 count>350/µl) for point-of-care CD4 tests.

POC-CD4 uses an Alere Pima point-of-care CD4 test (Alere, Waltham, Massachusetts, US). We estimate the point-of-care CD4 test's precision at 32.6% CV, as reported from a rural clinical care setting operated by non-research staff [36], and the test's bias at 0% (reported range, −12.3% to +16.5%) [36]–[38]. The point-of-care CD4 test has a sensitivity of 90% and a specificity of 85%, when ART eligibility is at CD4 count ≤250/µl (Figure 2A).

The laboratory CD4 test is estimated to cost US$10/test in Mozambique [39], whereas each point-of-care CD4 test costs US$24 [40]. Estimated test cost includes investments in equipment amortized over its usable lifetime, costs of materials to complete each test (including quality controls), and labor. We use the highest reported test cost from a microcosting approach [39],[40] to ensure that the analysis is not biased towards POC-CD4.

#### HIV clinical care

The Mozambique national guidelines inform inputs regarding patient monitoring, prophylaxis, and ART initiation at CD4 count ≤250/µl or a WHO stage 3 or 4 OI, including TB. Biannual laboratory CD4 tests monitor patient responses to ART; HIV RNA is not available [6]. Following ART initiation, first-line ART leads to virologic suppression in 79% of patients at 6 mo [29], resulting in rising CD4 counts [41]. Monitoring for ART failure uses immunologic criteria (e.g., ≥50% decrease in CD4 count or a CD4 count below nadir after ≥1 y of ART) [42] and prompts a switch to protease inhibitor–based second-line ART.

Estimates of loss to follow-up are from a systematic review of sub-Saharan African studies that excludes mortality [32]; we also correct for those patients who transfer care (i.e., not truly LTFU) [43]. The probability of LTFU is inversely related to adherence; patients with better ART adherence have lower LTFU rates (monthly probability, 0.2%) compared to patients with poorer adherence (monthly LTFU probability, 1.1%) [44]. Patients who are LTFU have a monthly probability of returning to care (1.0%) after being lost for 1 y [45] and return to care 50% of the time with a WHO stage 3/4 OI or TB. These input parameters result in 30.0%–34.8% of the cohort experiencing at least one interruption in HIV care that lasts 2.6 y (SD, 2.3 y) (Table 1). To assess the model's internal validity, we compare model output for LTFU at 36 mo with reported values from the published literature.

#### HIV care costs

Linked patients experience routine HIV care costs independent of initial immunologic staging strategy. We use costs of routine comprehensive HIV care for patients on ART ≤6 mo or >6 mo derived from 11 HIV treatment facilities in Mozambique, including the costs of clinical care, treatment and prophylaxis for OIs, and laboratory monitoring [46]. Given the model's structure, we use current CD4 count as a proxy for duration of ART (i.e., CD4 count ≤250/µl for ART ≤6 mo; CD4 count>250/µl for ART>6 mo). We assume that unlinked or LTFU patients incur only 20% of the costs of comprehensive HIV care. We evaluate the approach by comparing these estimated annual HIV care costs (US$160–US$250/y) with published primary data from countries with GDP and available HIV clinical care similar to those of Mozambique (reported range, US$115–US$338/y) [47]–[49]. Antiretroviral medication costs are from the Clinton Health Access Initiative (Table 1) [50]. All costs are converted to 2011 US dollars [26].

### Validation of the Model

We simulate HIV-uninfected individuals in the model to demonstrate its internal consistency.

### Deterministic Sensitivity Analyses

Guided by published literature, we perform one-way sensitivity analyses for overall linkage by individually varying the probability of test completion, receipt of CD4 test results, and initiation of care [15]–[17],[51], and point-of-care and laboratory test characteristics (see Tables S1–S3) [36]–[38],[52]–[56]. We vary CD4 test costs to capture the economies of scale associated with using one machine to complete more tests per day, as well as the lower labor costs in Mozambique and other sub-Saharan African countries [57]. We investigate cohort characteristics (e.g., age, gender, and mean CD4 count at the time of diagnosis) and features of clinical care (e.g., laboratory monitoring strategies, LTFU rates, and routine care costs) (Table 1).

### Scenario Analyses

#### Regional access to care

We consider the clinical outcomes and cost-effectiveness of POC-CD4 compared to LAB-CD4 in four different settings within sub-Saharan Africa to represent a range of access to repeat HIV testing, staging, and linkage to HIV care when ill with an acute OI or TB (Table 1).

#### ART eligibility at CD4 count ≤350/µl

To examine how expanded access to ART in Mozambique might affect the clinical and economic benefits of POC-CD4, we perform a scenario analysis in which national policy recommends initiation of ART at CD4 count ≤350/µl, such as in other sub-Saharan African countries. Using a CV of 32.6% and 0% bias [36], point-of-care CD4 tests have a sensitivity of 90% and specificity of 78% when ART eligibility is at CD4 count ≤350/µl (Figure 2B).

### Probabilistic Sensitivity Analysis

We perform a multi-way probabilistic sensitivity analysis to evaluate the effect of uncertainty around input parameters in the model and include all nine parameters for which one-way sensitivity analyses result in appreciable changes in ICERs. The model randomly selects a value for each parameter from a prespecified probability distribution (Table 2) and uses this combination of parameter values to calculate the expected clinical outcomes and costs for each strategy. The same process repeats 10,000 times for each strategy to obtain a distribution of outcomes and costs for each strategy. We first identify the more cost-effective strategy for each of the iterations and then assess the proportion of runs in which each strategy is identified as more cost-effective within a range of willingness-to-pay thresholds.

**Table 2:** Input parameters for probabilistic sensitivity analysis of immunological staging by POC-CD4 versus LAB-CD4 in Mozambique.

Variable	Distribution	Base Case Value	SD	Reference
Repeat HIV testing or immunologic staging (years)	Log-normal	10	4.8	Assumption
Cost per point-of-care CD4 test (US dollars)	Log-normal	24	6	[39],[40]
Overall linkage to care after POC-CD4 (percent)^a^	Beta	61	9.8	Adapted from [14]
Annual routine care costs ratio (percent)^b^	Log-normal	100	25	Adapted from [46]
Mean CD4 at diagnosis (cells/µl)	Log-normal	300	150	[14]
Point-of-care test CV (percent)^c^	Beta	32.6	10	[36]
Linkage after WHO stage 3 or 4 OI (percent)	Beta	75	12.5	Assumption
Mean age at diagnosis (years)	Log-normal	32.7	7.5	[14]
Linkage after TB (percent)	Beta	43	10	[33],[34]

a Overall linkage is varied using point-of-care test completion (83%, SD 13.5%).

b Annual routine care costs for patients with CD4 count ≤250/µl are US$250 (SD US$62.5), and for patients with CD4 count>250/µl are US$160 (SD US$40).

c Point-of-care test sensitivity (87%–95%) and specificity (76%–96%).

### Estimates of Uncertainty

We calculate the 95% confidence interval using model output for 5-y survival, costs, and life expectancy (undiscounted and discounted). We use Fieller's theorem to calculate the 95% confidence interval for the ICER [58],[59].

### Programmatic Considerations and Affordability

To investigate the affordability of POC-CD4, we assess the annual financial outlay associated with POC-CD4 compared to LAB-CD4 from the perspective of the Mozambique Ministry of Health (MMOH) and the donors who together provide funding for Mozambique's national response to the HIV/AIDS epidemic [60]. We consider the undiscounted direct costs of the two strategies for immunologic staging, as well as the costs of guideline-concordant HIV care incurred by those who link to care (Table 1). We include all equipment costs for immunologic staging in year one. We estimate that 120,000 people are newly diagnosed with HIV infection in the first year of the rollout of this strategy in Mozambique [61].

## Results

### Validation of the Model, Including Loss to Follow-Up

When the model simulates HIV-uninfected patients in Mozambique, life expectancy from birth is 54.7 and 58.3 y for males and females, respectively. This is consistent with WHO-reported overall life expectancy for Mozambique (i.e., 52.0 and 53.0 y, respectively), since the WHO estimates include HIV-infected people [62]. Of the simulated cohort, 10.6% are LTFU at 36 mo, which is consistent with estimates of 12.0% derived from published data [32],[43].

### Base Case

Five-year survival with LAB-CD4 is 60.9% (95% CI, 60.9%–61.0%), which increases to 65.0% (95% CI, 64.9%–65.0%) with POC-CD4 (Table 3). Discounted life expectancy is 9.6 y (95% CI, 9.6–9.6 y) with LAB-CD4 and increases to 10.3 y (95% CI, 10.3–10.3 y) with POC-CD4. Per person discounted lifetime costs are US$2,440 (95% CI, US$2,440–US$2,450) with use of LAB-CD4 and increase to US$2,800 (95% CI, US$2,790–US$2,800) with POC-CD4, which results in an ICER of US$500/year of life saved (YLS) in Mozambique. The 95% confidence interval surrounding the ICER is US$480–US$520/YLS, a value that has interpretable meaning in this situation because it reflects an unambiguous trade-off between costs and health outcomes [59],[63].

**Table 3:** Base case results of POC-CD4 versus LAB-CD4 for immunologic staging for HIV-infected persons in Mozambique.

Strategy	5-y Survival (Percent) (95% CI)	Lifetime Cost (US Dollars)		Life Expectancy (Years)		ICER (US Dollars/YLS) (95% CI)
		Undiscounted (95% CI)	Discounted (95% CI)	Undiscounted (95% CI)	Discounted(95% CI)	
LAB-CD4	60.9	3,930	2,440	14.0	9.6	—
	(60.9–61.0)	(3,920–3,940)	(2,440–2,450)	(14.0–14.0)	(9.6–9.6)	
POC-CD4	65.0	4,460	2,800	15.2	10.3	500
	(64.9–65.1)	(4,450–4,470)	(2,790–2,800)	(15.2–15.2)	(10.3–10.3)	(480–520)

### One-Way Sensitivity Analyses

**Figure 3:**
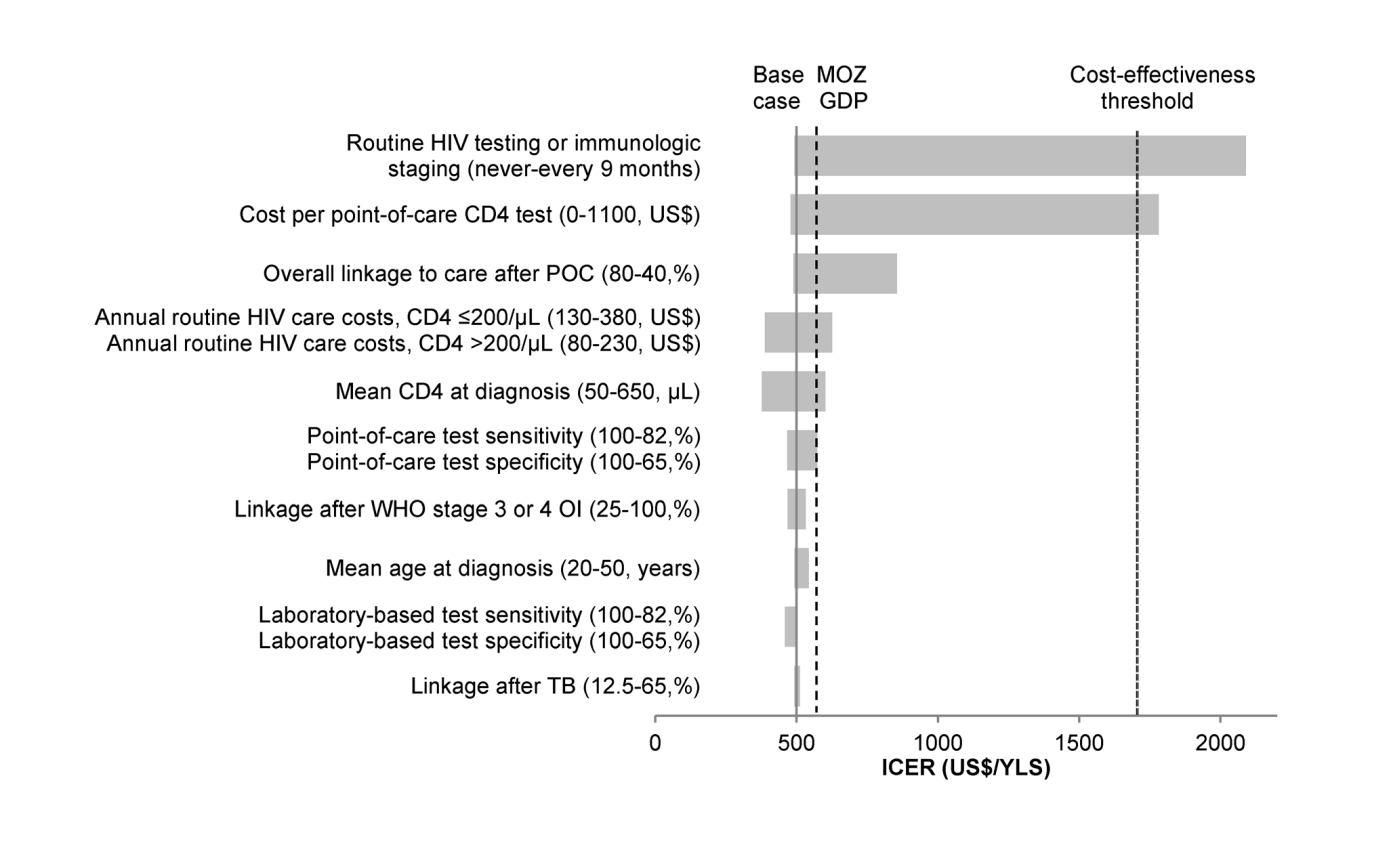
Tornado diagram of one-way sensitivity analyses when ART-eligibility threshold is at CD4 count ≤250/µl. A range of parameters varied in one-way sensitivity analyses are displayed on the vertical axis. The ICER (US dollars/YLS) of POC-CD4 compared to LAB-CD4 is represented on the *x*-axis. The solid vertical line indicates the ICER of the base case (US$500/YLS). The dashed vertical line represents the per capita GDP of Mozambique (MOZ GDP), i.e., the “very cost-effective” threshold; the dotted vertical line represents three times GDP, i.e., the “cost-effective” threshold. For each parameter, the horizontal bar represents the range of ICERs that result from varying that parameter along the range of values indicated in the parentheses; the first value listed in the parentheses is the one that results in the lowest ICER. Of all one-way sensitivity analyses, the ICER of POC-CD4 compared to LAB-CD4 crosses the cost-effectiveness threshold (into the area of not being cost-effective) (US$1,710/YLS) only when point-of-care CD4 test cost is>US$1,100/test or when repeat HIV testing or immunologic staging are completed every 9 mo or more frequently.

POC-CD4 results in improved clinical outcomes and remains cost-effective when compared to LAB-CD4 under a wide range of conditions in one-way sensitivity analyses (Figure 3). Clinical outcomes improve compared to LAB-CD4 as long as overall linkage increases with POC-CD4, which could result if test completion is ≥50%, receipt of results is ≥60%, or initiation of clinical care is ≥49% as a weighted average of ART-eligible and -ineligible patients. POC-CD4 remains cost-effective compared to LAB-CD4, even when the cost of the point-of-care test is far greater than currently reported. POC-CD4 is no longer cost-effective only when repeat HIV testing or immunologic staging for those patients who remain unlinked occurs every 9 mo or more frequently.

The benefits of earlier linkage and ART initiation with POC-CD4 might be attenuated if higher rates of loss to follow-up result after linkage with POC-CD4 compared with LAB-CD4. When the monthly probability of loss to follow-up is higher among patients who link to care via POC-CD4 (monthly probability, 0.02–0.003) than among those who link to care via LAB-CD4 (monthly probability, 0.01–0.002), then the percentage of the cohort who experiences one or more interruptions in care increases from 30.0% (LAB-CD4) to 45.5% (POC-CD4), and clinical outcomes are equivalent (discounted life expectancy, 9.6 y). When POC-CD4 includes an increased rate of loss to follow-up among ART-ineligible patients (monthly probability, 0.02 for all ART-ineligible patients with POC-CD4; therefore, 49.8% of patients experience LTFU), clinical outcomes are worse with POC-CD4 (discounted life expectancy, 9.4 y) compared to LAB-CD4 (discounted life expectancy, 9.6 y).

### Sensitivity Analysis for CD4 Test Characteristics

POC-CD4 results in better clinical outcomes and is cost-effective compared with LAB-CD4 even at reduced point-of-care test sensitivity (82%) and specificity (65%), which can result from either poor precision or extreme bias (Table 4). Such values are far below those published [36],[38],[52]. If the point-of-care CD4 test operates with perfect sensitivity and specificity, the ICER of POC-CD4 compared to LAB-CD4 is US$470/YLS, which reflects the increased rates of linkage with POC-CD4 and represents the cost-effectiveness ratio for ART in Mozambique. When laboratory CD4 tests operate with less precision or more bias (i.e., no longer a “perfect test”), then POC-CD4 becomes even more cost-effective compared to LAB-CD4 (ICER, US$460–US$500/YLS) (Figure 3).

**Table 4:** One-way and two-way sensitivity analyses of point-of-care CD4 test characteristics on misclassification of patients, clinical outcomes, costs, and cost-effectiveness compared to laboratory-based CD4 tests.

Analysis	CV (Percent)	Bias (Percent)	Sensitivity (Percent)^a^	Specificity (Percent)^a^	False Negatives (Percent)^b^	False Positives (Percent)^c^	Cost (US Dollars)^d^	Life Expectancy (Years)^d^	ICER (US Dollars/YLS)
**One-way sensitivity analysis on CV**	0		100	100	0.0	0.0	2,850	10.4	470
	5		98	98	2.1	1.6	2,830	10.4	470
	15		95	94	5.4	5.5	2,810	10.3	490
	25		92	89	8.0	10.8	2,800	10.3	500
	**32.6**		**90**	**85**	**9.6**	**15.1**	**2,800**	**10.3**	**500**
	45		88	79	11.9	21.2	2,790	10.2	520
	55		87	75	13.4	25.2	2,780	10.2	530
	70		85	70	15.3	29.6	2,770	10.2	550
	100		82	65	18.3	35.2	2,760	10.1	570
**One-way sensitivity analysis on bias**		−20	96	70	4.0	30.3	2,900	10.5	500
		−15	95	74	5.1	25.9	2,880	10.5	500
		−10	94	78	6.3	21.9	2,850	10.4	500
		−5	92	82	7.6	18.2	2,830	10.3	500
		**0**	**90**	**85**	**9.6**	**15.1**	**2,800**	**10.3**	**500**
		5	89	88	10.9	12.0	2,770	10.2	510
		10	87	90	12.8	9.6	2,750	10.2	510
		15	85	93	14.7	7.5	2,720	10.1	520
		20	83	94	16.7	5.8	2,700	10.1	540
**Two-way sensitivity analysis**	0.15	−20	99	78	0.6	21.7	2,900	10.5	480
	0.15	0	95	95	5.0	5.5	2,800	10.3	480
	0.15	20	84	99	15.7	0.5	2,690	10.0	520
	0.25	−20	98	73	2.5	26.9	2,910	10.5	490
	0.25	0	93	89	7.5	10.6	2,800	10.3	490
	0.25	20	84	97	16.1	3.0	2,690	10.1	530
	0.35	−20	95	69	4.6	31.3	2,900	10.5	500
	0.35	0	90	84	9.6	16.1	2,800	10.3	510
	0.35	20	83	93	16.9	6.8	2,700	10.1	540

Base case in bold.

a Derived using test performance of percent SD  = 32.6% [36], mean CD4 count (SD) of cohort  = 300/µl (230/µl), and ART-eligibility threshold at CD4 count ≤250/µl,

b False positives: patients who are ART ineligible (true CD4 count>250/µl) but are misclassified as ART eligible.

c False negatives: patients who are ART eligible (true CD4 count ≤250/µl) but are misclassified as ART ineligible.

d Discounted at 3%/y.

### Probabilistic Sensitivity Analysis

When varying nine parameters based on their prespecified probability distributions (Table 2), POC-CD4 is more cost-effective than LAB-CD4 92% of the time at the willingness-to-pay threshold of US$570/YLS, or the Mozambique 2011 per capita GDP. The probability of POC-CD4 being more cost-effective than LAB-CD4 is even greater at higher willingness-to-pay thresholds.

### Scenario Analyses

#### Regional access to care

**Figure 4:**
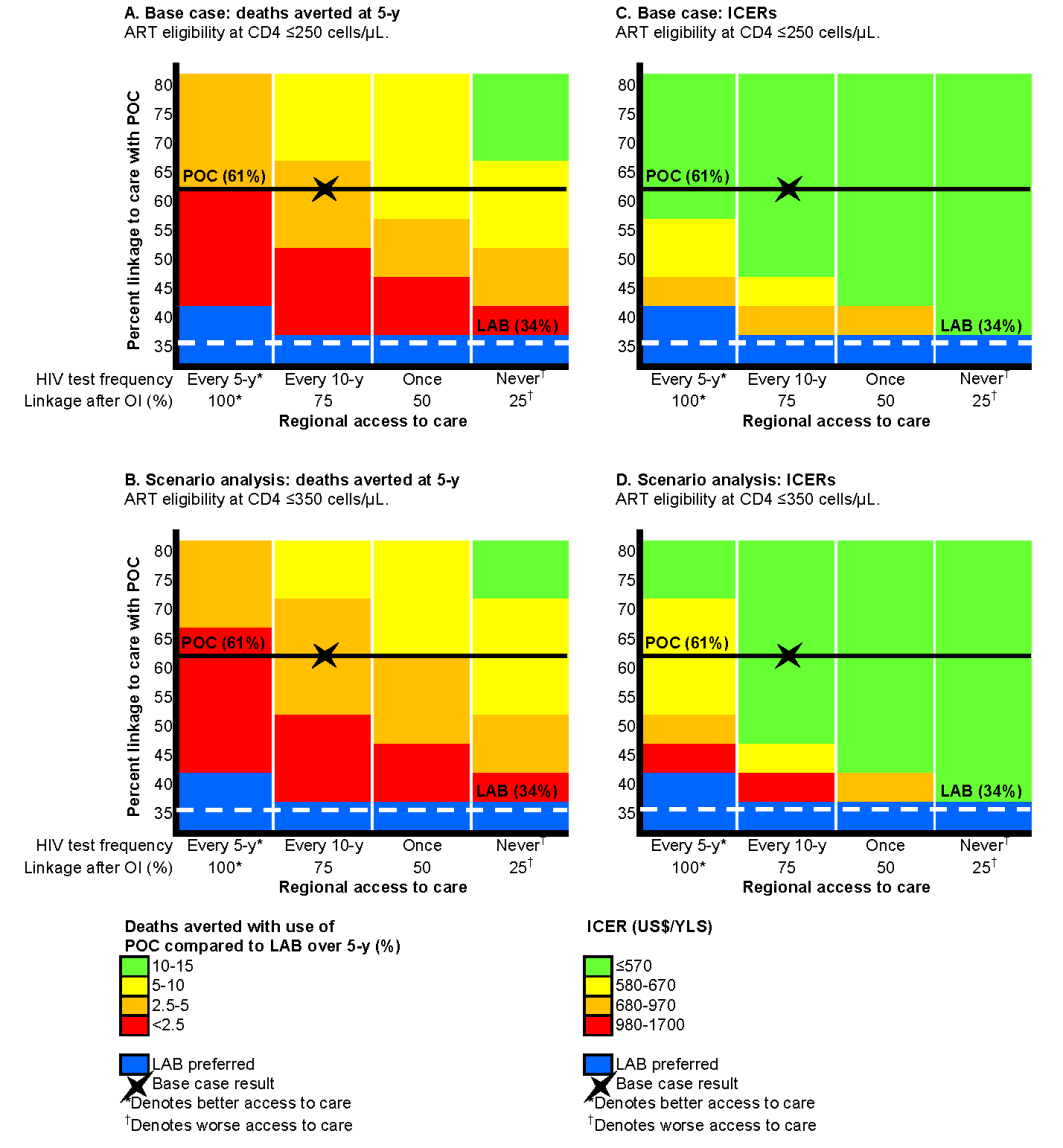
Multi-way sensitivity analysis on regional access to care and linkage to care with POC-CD4 compared to LAB-CD4. Projected decrease in 5-y mortality and ICERs with use of POC-CD4 compared to LAB-CD4 in four settings that represent a range of access to care (decreasing access to care from left to right) and with different probabilities of linkage with POC-CD4 (increasing up the vertical axis). The base case is indicated by the X in each figure; the horizontal lines represent the base case overall linkage rates (POC-CD4, solid black; LAB-CD4, dashed white). Decreased mortality at 5 y with POC-CD4 is projected in settings that use current Mozambique guidelines for ART eligibility (CD4 count ≤250/µl) (A) or earlier ART eligibility (CD4 count ≤350/µl) (B). More deaths could be averted (noted by changes in color towards green) in settings with fewer opportunities to access care or by improving POC-CD4 linkage rates compared to LAB-CD4. Blue denotes the few situations in which LAB-CD4 results in better clinical outcomes than POC-CD4 at 5 y. (C) displays the ICERs of POC-CD4 compared with LAB-CD4 given current Mozambique guidelines (ART eligibility at CD4 count ≤250/µl); (D) displays ICERs in settings with earlier ART eligibility (CD4 count ≤350/µl). POC-CD4 is at the very cost-effective threshold (i.e., US$450–US$860/YLS in [C] and US$460–US$1,030/YLS in [D]) compared to LAB-CD4 except when linkage with POC-CD4 is <5% better than LAB-CD4 in settings with repeat HIV testing every 5 y.

In settings where access to care provides fewer opportunities to test and link to care, POC-CD4 leads to a greater increase in 5-y survival than LAB-CD4. For instance, as shown in Figure 4A, 2.3% of deaths (red) are averted at 5 y with POC-CD4 compared to LAB-CD4 in a setting with greater access (Figure 4A, far left column), but 8.1% of deaths (yellow) are averted if POC-CD4 is used in a setting with less access to care (Figure 4A, far right column). The clinical benefits of POC-CD4 increase as POC-CD4 linkage improves, reflected in an increased percentage of deaths averted (Figure 4A, ascending the vertical axis). Where regional access to care is less robust, POC-CD4 becomes more cost-effective compared to LAB-CD4, as long as linkage to care with POC-CD4 is better than with LAB-CD4 (Figure 4C). POC-CD4 ceases to be clinically preferred or cost-effective compared to LAB-CD4 only when POC-CD4 improves linkage by <5% in settings where repeat HIV testing occurs at least every 5 y and diagnosis with an OI always leads to linkage to HIV care (Figure 4A and 4C, far left column).

#### ART eligibility at CD4 count ≤350/µl

At an alternative ART-eligibility threshold of CD4 count ≤350/µl, 5-y survival increases to 61.5% with use of LAB-CD4 and 65.1% with POC-CD4. The discounted life expectancy increases to 9.8 y with LAB-CD4; POC-CD4 further improves life expectancy to 10.4 y. Per person discounted lifetime costs are also greater with the alternative ART-eligibility threshold (LAB, US$2,570; POC, US$2,900), resulting in an ICER of US$530/YLS for POC-CD4 compared to LAB-CD4. As linkage after point-of-care CD4 tests improves, POC-CD4 is clinically preferred and more cost-effective compared to LAB-CD4 in settings where access to repeat testing and linkage to care is less available (Figure 4B and 4D).

### Programmatic Considerations and Affordability

For the 120,000 newly diagnosed patients in Mozambique in 1 y, we estimate that the MMOH and other donors would pay US$600,000 for immunologic staging with LAB-CD4, which would increase to US$2,400,000 with POC-CD4. Taking into account the undiscounted costs of follow-up HIV care for those patients who successfully link, the MMOH and other donors would fund US$78.2 million for LAB-CD4 compared to US$94.1 million for POC-CD4 over 5 y. The costs associated with immunological staging itself are a very small proportion of the overall 5-y costs (LAB-CD4 0.8%; POC-CD4 2.5%); most of the increased costs are related to clinical care and ART for the patients who link to care.

## Discussion

Using a simulation model of HIV disease, we find that a POC-CD4 strategy of immunological staging results in nearly one full year of additional life expectancy compared to LAB-CD4 and is near the very cost-effective threshold in Mozambique. To remain conservative regarding any benefit of POC-CD4, we use the published estimates of point-of-care CD4 test characteristics and costs that are among the least favorable. The value of POC-CD4 compared to LAB-CD4 will likely be even greater if point-of-care CD4 tests operate with improved test characteristics [53] or lower cost [39], or if laboratory-based CD4 tests operate with less than perfect test characteristics. The majority of costs are due to the clinical care provided after linkage to care; as the costs of care decrease, the relative value of POC-CD4 increases compared to LAB-CD4.

POC-CD4 results in better clinical outcomes and is at the very cost-effective threshold compared to LAB-CD4 if POC-CD4 leads to enhanced linkage; much of this improvement is due to increased test completion and receipt of results. Attention must therefore remain focused on sustaining improvements in any of the sequential steps of linkage after POC-CD4 [15],[64]. Settings in sub-Saharan Africa with robust transport and centralized laboratory systems already in place might invest to improve LAB-CD4 as an alternative approach for improving patient outcomes. However, the costs of improving transport and infrastructure could well outpace the costs associated with POC-CD4, which could also be implemented more quickly to assist in rapid scale-up.

Although performance characteristics should be a high priority with any new diagnostic test, our analysis suggests that the impact of point-of-care CD4 tests on linkage outweighs the effect of the tests' performance characteristics within reasonable ranges. Because the goal of immunological staging is to expedite linkage to care for those most in need, a POC-CD4 strategy with some misclassified test results can still lead to improved clinical outcomes, if overall linkage is better than with LAB-CD4 and if ART programs can incorporate new ART-eligible patients promptly.

Our results hold in a diversity of conditions. The more expensive POC-CD4 strategy still offers excellent value under a wide range of plausible scenarios that represent a variety of settings in sub-Saharan Africa, including a range of linkage rates, opportunities for subsequent access to care, and loss to follow-up. However, this value is realized only if ART is available for those patients who link to care, if patients remain in care, and if sufficient and sustainable funds are available for a lifetime of clinical care.

As with many cost-effectiveness models, there is a fundamental assumption about what constitutes cost-effective care. We apply the WHO-CHOICE standard that uses per capita GDP as a threshold. Mozambique stands in the lowest tertile of per capita GDP reported in sub-Saharan Africa (US$570; range, US$220–US$12,400). In considering the generalizability of these results to other sub-Saharan countries with greater capacity to pay for lifesaving care (e.g., Angola or Botswana, with per capita GDPs of US$5,300 and US$9,500, respectively), POC-CD4 will likely remain an attractive option over an even wider range of input value assumptions.

Despite its good value, POC-CD4 is not without cost. We estimate an additional US$1,800,000 due to point-of-care CD4 tests in the first year of use, if 120,000 patients are eligible for immunological staging. An overwhelming majority of the increased costs associated with POC-CD4 over 5 y (88.7% of the US$15.9 million) are due not to the test itself, but rather to the downstream costs (e.g., ART and clinical visits) associated with an increased number of people engaged in HIV clinical care. Such costs would be incurred for any patients who link to care, regardless of method of linkage (e.g., POC-CD4). Given the annual budget of more than US$146 million for HIV care funded by Mozambique and donors [60], our analysis suggests that POC-CD4 is a feasible option because the increased associated costs represent 2.2% of what is currently spent on the AIDS response in Mozambique.

While some argue that the most resource-constrained settings cannot afford the cost of POC-CD4, our results indicate that this is likely where the greatest value lies. Our findings support the use of POC-CD4 particularly in settings where alternative opportunities for linkage to care are limited or other interventions have failed. The comparative value of POC-CD4 compared to LAB-CD4 at VCT clinics may be less if other strategies are also used to enhance subsequent linkage to HIV care after an initial failure to link, including home-based testing and linkage to care [65], mobile clinics [66], mHealth technologies [67], peer navigators [68]–[70], or decentralization of HIV clinical care [71]. Our results underscore that the opportunity to access care after HIV diagnosis is an important indicator of resource limitation and could guide where POC-CD4 implementation would be of greatest value. According to the nationwide registry of CD4 testing in the national health service (I. V. J.), 22% of CD4 tests in the public sector are now being performed using POC-CD4 in Mozambique, with site selection focused on areas with poor access to existing laboratories. Because each country in sub-Saharan Africa includes a diversity of settings for access to care, the value of POC-CD4 implementation could be maximized by targeting specific settings where opportunities for subsequent linkage are least available.

This analysis has several limitations. We do not address the use of point-of-care CD4 tests for routine monitoring [72]. In cases where point-of-care testing will repeatedly influence clinical management, poor test characteristics and increased cost of a point-of-care test could have a greater impact on clinical outcomes and lifetime costs. Although POC-CD4 costs include estimates for labor, quality control, etc., a more comprehensive rollout of POC-CD4 could reveal additional operational challenges such as instrument or operator failure, which could further reduce the efficacy or increase the costs of POC-CD4 compared to LAB-CD4. In our simulation, we do not directly assess the POC-CD4 impact of increasing ART coverage on reducing HIV transmissions [73]. However, the incorporation of any decreased transmissions due to earlier ART initiation resulting from POC-CD4 into this model-based analysis would further increase the value of POC-CD4 compared with LAB-CD4.

Too many eligible patients still await ART initiation. It is important to identify cost-effective methods for immunologic staging that will expedite access to care for the high-priority cases of the most immunosuppressed individuals throughout sub-Saharan Africa. Point-of-care CD4 tests are now available, and a growing body of evidence supports improved overall linkage to care with their use. We find that a POC-CD4 strategy can avert deaths and offers excellent value for immunologic staging compared to LAB-CD4 across a wide range of parameters in Mozambique, as well as in a diversity of resource-limited settings. Despite a modest increase in costs, POC-CD4 could remain economically efficient and have the greatest impact on mortality in settings throughout sub-Saharan Africa, where health care resources and systems are the most limited.

## Supporting Information

Table S1 Range of reported bias for Alere Pima point-of-care CD4 tests compared to laboratory CD4 tests.
(DOCX)

Table S2 Range of sensitivity and specificity for Alere Pima point-of-care CD4 tests determining ART eligibility at different thresholds compared to laboratory CD4 tests.
(DOCX)

Table S3 Range of misclassification by Alere Pima point-of-care CD4 tests regarding ART eligibility at different thresholds compared to laboratory CD4 tests.
(DOCX)

## Abbreviations

ART: antiretroviral therapy
CEPAC-I: Cost-Effectiveness of Preventing AIDS Complications–International
CV: coefficient of variation
GDP: gross domestic product
ICER: incremental cost-effectiveness ratio
LAB-CD4: laboratory-based CD4 testing
LTFU: lost to follow-up
MMOH: Mozambique Ministry of Health
OI: opportunistic infection
POC-CD4: point-of-care CD4 testing
SD: standard deviation
TB: tuberculosis
VCT: voluntary testing and counseling
WHO: World Health Organization
YLS: year of life saved
