# Supplementary material for: The Clinical and Economic Impact of Point-of-Care CD4 Testing in Mozambique and Other Resource-Limited Settings: A Cost-Effectiveness Analysis
Source: PLoS Med. 2014 Sep 16;11(9):e1001725. doi: 10.1371/journal.pmed.1001725 (PMC4165752; doi:10.1371/journal.pmed.1001725)
Supplement: Table S1 — Range of reported bias for Alere Pima point-of-care CD4 tests compared to laboratory CD4 tests. (DOCX) [file pmed.1001725.s001.docx]

**Table S1: Range of reported bias for Alere Pima point-of-care CD4 test compared to laboratory CD4 tests.**

|  | **Mean bias (%)** |
| --- | --- |
| Sensitivity analysis | -20.0 to 20.0 |
| Jani et al. *AIDS* 2011 | -12.3 |
| Sukapirom et al. *JAIDS* 2011 | -10.8 |
| Glencross et al. *JIAIS* 2012 | +16.5 |
