# Supplementary material for: The Clinical and Economic Impact of Point-of-Care CD4 Testing in Mozambique and Other Resource-Limited Settings: A Cost-Effectiveness Analysis
Source: PLoS Med. 2014 Sep 16;11(9):e1001725. doi: 10.1371/journal.pmed.1001725 (PMC4165752; doi:10.1371/journal.pmed.1001725)
Supplement: Table S2 — Range of sensitivity and specificity for Alere Pima point-of-care CD4 tests determining ART eligibility at different thresholds compared to laboratory CD4 tests. (DOCX) [file pmed.1001725.s002.docx]

**Table S2: Range of sensitivity and specificity for Alere Pima point-of-care CD4 test determining ART-eligibility at different thresholds compared to laboratory CD4 tests.**

|  | **ART-threshold**  **≤250/μL** | | **ART-threshold**  **≤300/μL** | | **ART-threshold**  **≤350/μL** | |
| --- | --- | --- | --- | --- | --- | --- |
|  | **Se (%)** | **Sp (%)** | **Se (%)** | **Sp (%)** | **Se (%)** | **Sp (%)** |
| Sensitivity analysis | 82-100 | 65-100 |  |  | 80-100 | 61-100 |
| Mnyani et al. J*AIDS* 2012 | 93 | 86 |  |  |  |  |
| Manabe et al. *PLoS One* 2012 | 96 | 87 | 93 | 80 |  |  |
| Diaw et al. *JAIDS* 2011 |  |  |  |  | 91 | 96 |

ART: antiretroviral therapy; Se: sensitivity; Sp: specificity.
