# Supplementary material for: The Clinical and Economic Impact of Point-of-Care CD4 Testing in Mozambique and Other Resource-Limited Settings: A Cost-Effectiveness Analysis
Source: PLoS Med. 2014 Sep 16;11(9):e1001725. doi: 10.1371/journal.pmed.1001725 (PMC4165752; doi:10.1371/journal.pmed.1001725)
Supplement: Table S3 — Range of misclassification by Alere Pima point-of-care CD4 tests regarding ART eligibility at different thresholds compared to laboratory CD4 tests. (DOCX) [file pmed.1001725.s003.docx]

**Table S3: Range of misclassification by Alere Pima point-of-care CD4 test regarding ART-eligibility at different thresholds compared to laboratory CD4 tests.**

|  | **ART-threshold**  **≤200/μL** | | **ART-threshold**  **≤250/μL** | | **ART-threshold**  **≤350/μL** | |
| --- | --- | --- | --- | --- | --- | --- |
|  | **FN (%)** | **FP (%)** | **FN (%)** | **FP (%)** | **FN (%)** | **FP (%)** |
| Sensitivity analysis |  |  | 0 – 18.3 | 0 – 35.2 | 0 – 19.6 | 0 – 39.1 |
| Jani et al. *AIDS* 2011 | 0 | 5.2 |  |  | 2.2 | 14.8 |
| Mtapuri-Zinyoera et al. *JAIDS* 2010 | 2.4 | 4.2 |  |  | 4.2 | 2.4 |

ART: antiretroviral therapy; FN: false-negative; FP: false-positive.
